# Effects of pneumatic tube systems on viscoelastic coagulation tests in septic patients and healthy individuals: protocol of the randomized controlled VETaPT-trial

**DOI:** 10.3389/fmed.2025.1613276

**Published:** 2025-06-10

**Authors:** Martin Mirus, Erik Buehrer, Lars Heubner, Christian Schnabel, Thea Koch, Peter Markus Spieth

**Affiliations:** Department of Anaesthesiology and Intensive Care Medicine, Faculty of Medicine and University Hospital Carl Gustav Carus, TUD Dresden University of Technology, Dresden, Germany

**Keywords:** viscoelastometry, coagulation, sepsis, platelet function, pneumatic tube system

## Abstract

**Introduction:**

Rapid coagulation assessment is crucial in emergencies, especially with acute bleeding, where timely intervention prevents shock and circulatory failure. Viscoelastic tests (VET) offer real-time insights into clot formation, fibrinolysis, and overall coagulation dynamics, often surpassing conventional lab tests. To accelerate diagnostics, hospitals use pneumatic tube systems (PTS) for blood transport. However, the effect of PTS transport on VET results remains unclear, especially with newer VET technologies and in critically ill patients, who may be more vulnerable to acceleration forces.

**Objective:**

The VETaPT (Viscoelastic Testing after Pneumatic Tube Transport) trial investigates whether PTS transport influences results of three next generation VET and platelet function testing in healthy volunteers and critically ill septic patients. It explores if acceleration during transport alters coagulation and platelet function parameters and whether septic patients exhibit increased susceptibility due to their altered coagulation profiles. A goal is to define an acceleration threshold above which PTS-related effects become clinically relevant. This threshold could allow assessment of transport suitability based on force data alone, supporting wider clinical application without repeated blood testing.

**Study design:**

This prospective, randomized clinical trial includes both healthy volunteers and critically ill septic patients. Paired blood samples are collected and randomly assigned to either manual or PTS transport, with each subject serving as their own control. Acceleration forces during PTS transport are continuously recorded using a three-axis accelerometer. Samples are analyzed using standard lab tests and following point-of-care devices: ClotPro^®^, ROTEM^®^ sigma, Multiplate^®^, and TEG6s^®^. The primary outcome is the difference in coagulation parameters between transport methods, evaluated in the context of measured acceleration forces.

**Expected results:**

This is the first study to systematically compare multiple next generation VET and platelet aggregation systems in both healthy and septic patients under controlled PTS transport conditions. It is hypothesized that PTS-induced acceleration may alter test results. Identifying a critical threshold could ensure safe, rapid blood transport without compromising diagnostic quality, potentially reducing personnel needs and expediting therapy initiation.

**Registration:**

Ethics approval was obtained from the responsible committee of the Technical University Dresden (BO-EK-12012024_1). The study is registered with the German Clinical Trials Register (DRKS00036231).

## 1 Introduction

Rapid analysis of blood samples can be life-saving in time-critical emergency situations. Acute bleeding is one such scenario, requiring immediate intervention to prevent shock caused by hypoxia and hypoperfusion, which may lead to circulatory failure. The assessment of coagulation parameters plays a crucial role in guiding therapeutic decisions, such as the administration of blood products or coagulation factor treatments. To facilitate prompt intervention, these parameters must be available as quickly as possible. The development of point-of-care (POC) devices for viscoelastic testing (VET) represents a significant advantage in emergency diagnostics, enabling rapid coagulation assessment. In addition to their speed, VET devices provide valuable insights into coagulation dynamics, which are neglected in standard laboratory tests (SLT), such as INR/Quick, aPTT, platelet counts, and fibrinogen levels (1–4). Traditional tests fail to account for key cellular components of coagulation (e.g., platelets and erythrocytes), the interaction of coagulation factors, and clot resolution (fibrinolysis), all of which play critical roles in hemostasis. Under physiological conditions, clot formation and fibrinolysis are balanced, but critically ill patients may experience excessive clot breakdown (hyperfibrinolysis) or impaired clot resolution (hypofibrinolysis), leading to bleeding complications or thromboembolic events (4, 5). VET POC devices offer real-time insights into fibrinolysis, surpassing the imprecise estimations provided by SLT. By enabling faster and more targeted diagnostics, VET devices facilitate timely interventions, potentially reducing the need for blood products and improving patient outcomes (6). However, decentralized access to VET devices is not universal, making rapid transport to central VET units essential for timely results. Pneumatic tube systems (PTS) are widely used in hospitals to expedite sample transport and reduce staff workload (7). The potential impact of acceleration forces in the PTS on the VET results has been debated since the early days of these devices (8–11). However, findings remain inconsistent (12, 13), and it is unclear whether older studies apply to next generation VET devices, as manufacturers continue to develop models with increasingly diverse technical approaches (2, 14, 15). Recent developments in VET devices include platelet function analysis (16) and assessments of hypo-and hyperfibrinolysis (2). While early VET devices relied on mechanical detection of coagulation activation, newer models also incorporate acoustic measurements on the basis of the clot resonance frequency (14). This, in turn, raises new questions. Namely, whether external vibrations might influence the results of VET measurements. Indeed, Zipperlein et al. demonstrated that vibrations during helicopter transport can affect VET outcomes (17). This growing diversity complicates direct comparisons between devices and makes it difficult to predict how acceleration forces during PTS transport might affect VET results. Studies have shown that PTS transport can induce hemolysis in blood samples (18) and alter coagulation parameters (10), likely due to shear forces acting on erythrocytes and platelets. Acceleration forces are likely more relevant to shear stress than is the sustained velocity (19, 20). It is well established that mechanical forces can alter blood components even *in vivo*. For example, high shear stress in aortic valve stenosis can degrade von Willebrand factor, leading to acquired von Willebrand syndrome (21). These forces may activate coagulation factors and platelets, but the findings remain inconsistent (19, 20). Discrepancies in previous studies may arise from differences in blood sample characteristics. Blood from healthy individuals may respond differently to acceleration forces than blood from critically ill patients with altered coagulation profiles. This variation complicates comparisons between studies using healthy volunteers versus patient samples (9, 10, 22, 23). Another limitation is that most studies rely solely on manufacturer-reported transport speeds (11), while others report only maximum velocities without further analyzing the specific effect of acceleration forces during PTS transport on test parameters (8–10, 24). These approaches fail to consider the following critical issues:

- Does the actual transport speed match the manufacturer’s specifications?
- How long is the blood sample exposed to this speed?
- What acceleration forces occur at the start and end of transport?
- Is there a correlation between the magnitude of the acceleration forces and alterations influence in POC parameters?
- Is the observed effect clinically meaningful, or is it merely statistically significant?

Therefore, precise quantification of the forces acting within a PTS is essential for understanding their potential impact on blood samples. This study hypothesizes that transport of blood samples via a PTS has a clinically significant impact on results obtained from next generation VET devices. This effect is expected to correlate with the magnitude of acceleration forces during transport and may differ between healthy individuals and critically ill septic patients due to sepsis-associated coagulation abnormalities.

## 2 Methods and analysis

This trial protocol adheres to the SPIRIT guidance for protocols of clinical trials (25).

### 2.1 Trial objectives

The Viscoelastic Testing after Pneumatic Tube Transport (VETaPT) trial study investigates whether PTS transport has a clinically significant impact on coagulation results obtained from of three next generation POC VET devices and platelet function testing in healthy volunteers and critically ill septic patients. The primary outcome is the difference in test results between manually and PTS-transported blood samples in both healthy volunteers and critically ill septic patients.

As secondary objectives, the study explores whether the magnitude of acceleration forces during PTS transport correlates with changes in coagulation parameters and whether this effect differs between healthy and septic individuals due to sepsis-associated coagulation alterations. In addition, the study aims to define a threshold of PTS-induced forces beyond which clinically relevant deviations in test results occur. The study’s area under the curve (AUC)-based method may enable the findings to be extrapolated to other PTS systems without requiring further blood sample transport and analysis. Such a threshold could help establish safe transport conditions and enable the generalization of results to other PTS systems using force measurements alone, without the need for additional blood sample testing.

### 2.2 Trial design

**Figure 1:**
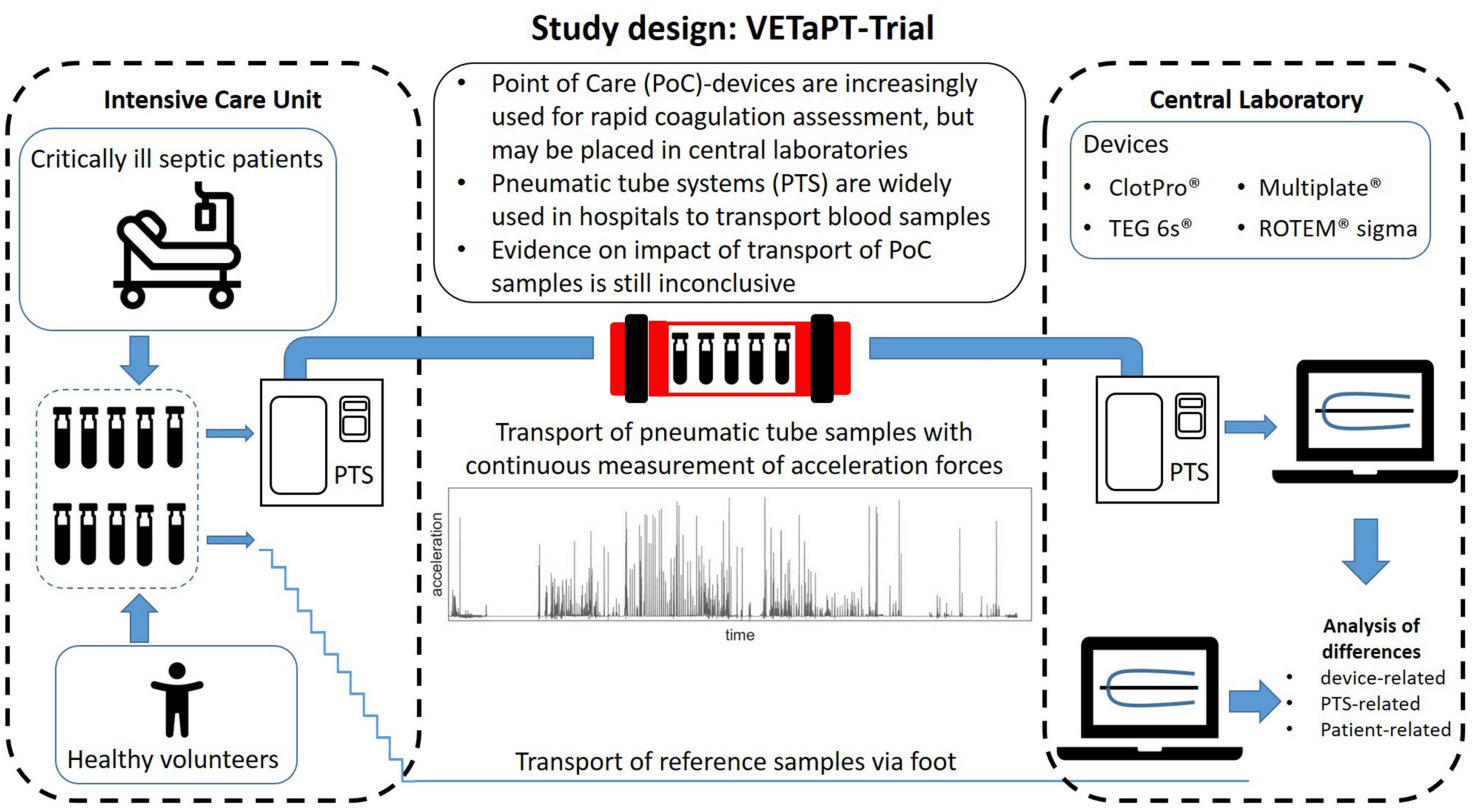
Study Design of the VETaPT trial. Blood samples from both healthy volunteers and critically ill septic patients are collected for both manual and PTS transport and randomly assigned to a transport modality. During PTS transport, acceleration forces are continuously monitored via a three-axis accelerometer. For the investigation, blood samples will be analyzed with both standard laboratory tests and four point-of-care devices. PTS: Pneumatic tube system; POC: Point-of-care.

This prospective, randomized clinical study will evaluate the effects of PTS transport on coagulation in both healthy volunteers and critically ill septic patients. Following enrolment and informed consent, blood samples will be collected from each participant. For each participant, samples for both manual and PTS transport will be drawn simultaneously and assigned randomly. An overview of the study design is provided in Figure 1. Since each participant serves as their own control, no additional control group or participant randomization is needed. Acceleration forces during PTS are continuously measured during each transport cycle via a three-axis accelerometer.

### 2.3 Study population and recruitment

Participant selection is based on predefined inclusion criteria and is conducted entirely on a voluntary basis, with informed consent obtained before participation.

Inclusion criteria

Healthy volunteers:

- ≥ 18 years
- No acute illnesses or known chronic diseases affecting coagulation
- No medication intake within the last 7 days
- No known pregnancy
- Written informed consent was provided
  Critically ill septic patients:
- ≥ 18 years
- Requiring intensive care with a Sequential Organ Failure Assessment (SOFA) score of ≥ 9
- Patients were diagnosed with sepsis or septic shock within the last 48 h according to the SEPSIS-3 criteria (26).
- Undergoing anticoagulation therapy with unfractionated heparin (UFH), low molecular weight heparin (LMWH), or argatroban at any dosage
- Written informed consent by patient or legal representative
  Exclusion Criteria
- Known hereditary coagulation

For recruitment, critically ill patients will be screened and allocated from the intensive care unit (ICU) of the Department of Anesthesiology an Intensive Care Medicine at the University Hospital Dresden. Healthy volunteers will be recruited via announcements at the medical faculty of the study center.

### 2.4 Setting and intervention

**Figure 2:**
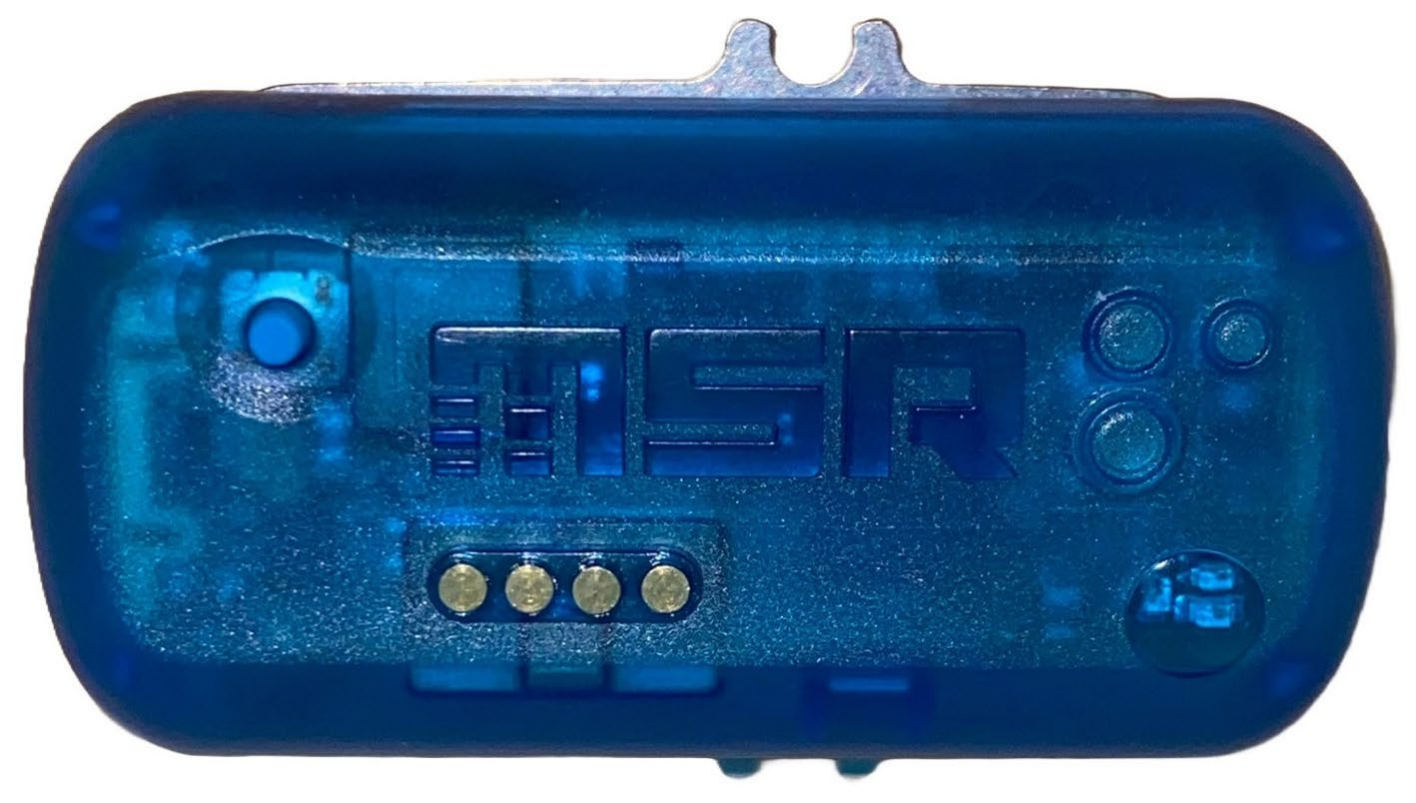
Data logger. This three-axis accelerometer (MSR 145, MSR Electronics, Switzerland) enables the measurement of acceleration along three spatial axes with a sampling rate of 1 kHz, storing the highest recorded value at 50 Hz. Owing to its lightweight and miniaturized design, the sensor can be easily transported within the PTS, and acceleration forces can be continuously monitored during PTS transport.

Coagulation tests will be performed according to standard clinical procedures. To minimize interindividual variability, all participants’ blood samples will be analyzed both after manual transport and after automated transport via PTS. Each participant serves as their own control. To avoid artifacts that could arise from transferring a portion of the blood from one sample tube into another (e.g., shear forces or bubble formation due to incomplete tube filling), which does not reflect clinical reality, separate blood samples will be drawn for PTS and manual transport for all participants. Blood samples will be collected simultaneously for each participant, and the transport modality (manual or PTS) for specific samples will be randomized. To address these two research questions, blood samples will be analyzed after manual transport and after pneumatic tube transport. During transport, speed and acceleration are continuously recorded by a data logger (accelerometer, Figure 2) that is transported along with the blood sample. This enables the assessment of the variability of the acceleration forces within the PTS, despite the constant nominal speed and identical transport routes. Since blood samples are transported at different times and days, variations in PTS utilization and transport dynamics (e.g., delays, intermittent stops) naturally occur, which introduces random variations in the forces acting during transport. Additionally, alternative speeds will be programmed within the PTS to introduce controlled variations in acceleration forces to investigate the threshold at which PTS forces affect coagulation analyses. There are also two possible pathways between the ICU and the central laboratory in the study center once more variations are generated. To conclude, the following systematic variations in PTS transport conditions will be implemented in the VETaPT trial: 1. High vs. low transport speed by adjusting the PTS compressor settings; 2. Transport of blood samples in a small transport bag vs. in a specific insert that fixates the samples within the PTS container. The decision regarding the use of the insert or a bag as well as the speed settings is made randomly before the start of automated transport. This will further increase the variability of the AUC value as a parameter of the applied accelerations.

For five participants, a vibration exciter (LDS Permanent Magnet Vibration System, V455 Series, Hottinger Brüel & KJÆR GmbH) will be used to generate controlled acceleration forces, allowing precise evaluation of their effect on coagulation results. Unlike the PTS, which produces varying acceleration magnitudes, the vibration exciter enables precise and reproducible acceleration forces. This approach is expected to provide more precise insights into which components of the AUC may have a disproportionately high impact on possible changes in the test results.

### 2.5 Relevance for critically ill septic patients

Septic patients often exhibit altered coagulation profiles (4, 5, 27), making it uncertain whether findings from healthy volunteers can be directly extrapolated to this population. Since it is unknown whether the known coagulation system alterations described in critically ill patients influence their susceptibility to PTS-induced forces, the VETaPT trial will include both healthy volunteers and critically ill septic patients. Furthermore, since critically ill patients often require rapid laboratory diagnostics, it is particularly important to investigate potential PTS transport effects in this group. During routine clinical blood sampling, additional blood samples will be collected for study purposes. These will be analyzed after both manual and PTS transport, ensuring that each patient serves as their own control.

### 2.6 Quantifying acceleration forces

**Figure 3:**
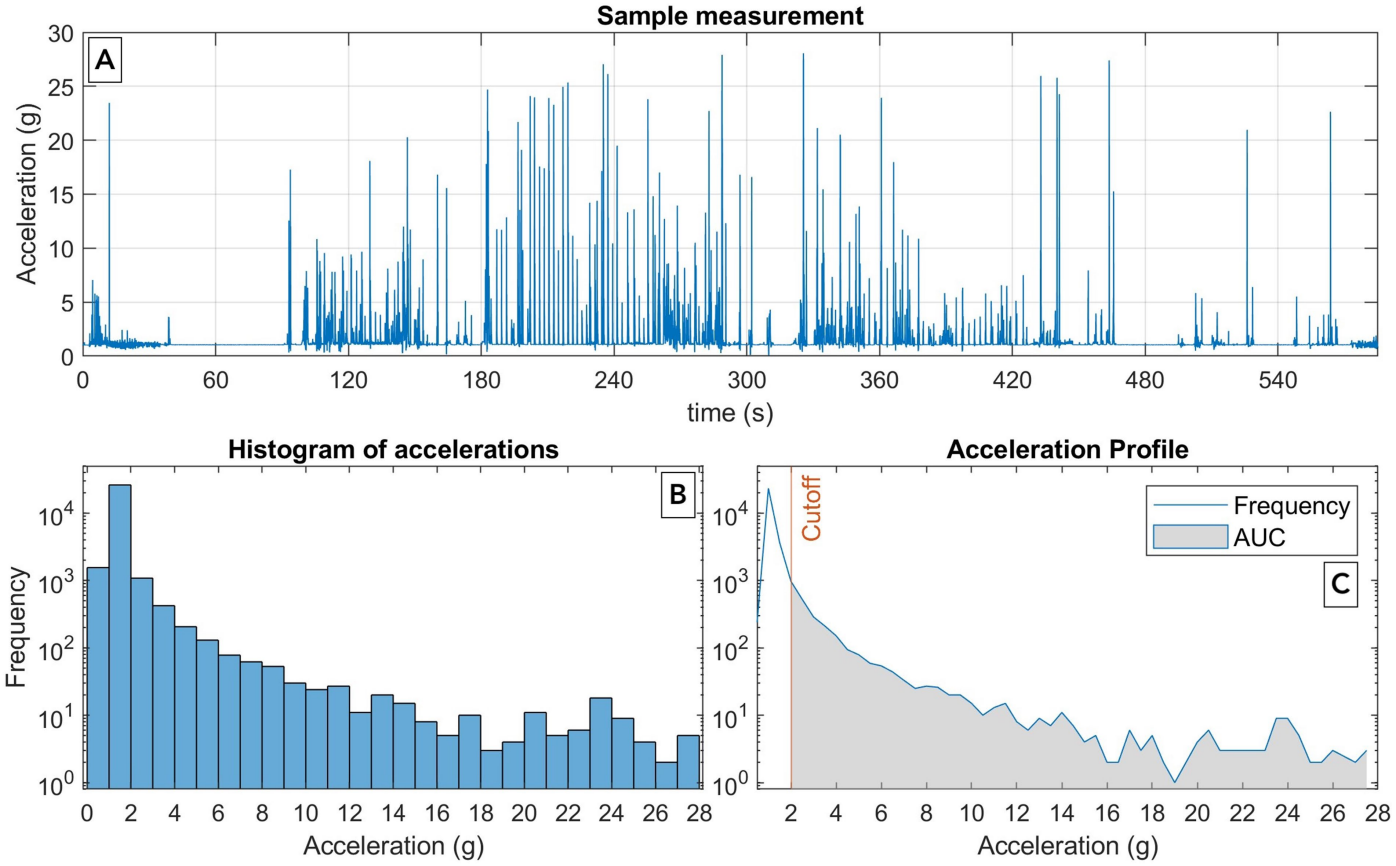
Quantification of acceleration forces during pneumatic tube transport. **(A–C)** Illustrates the quantification of acceleration forces for one transport process via an area under the curve (AUC)-based method. Digital analysis of the recorded data from the data logger enables visualization of acceleration over the transport duration **(A)**. In the next step, the absolute vector values are categorized into bins, similar to a histogram **(B)**. The number of bins has been decreased for illustration purposes. The values exceeding a predefined acceleration threshold, defined as the typical acceleration occurring during manual transport, are subsequently summed **(C)**. This approach allows the forces acting during transport to be graphically represented as the AUC value for each transport instance **(C)**.

During transport, speed and acceleration are continuously recorded via a data logger. For this purpose, a three-axis accelerometer (MSR 145, MSR Electronics, Switzerland) (Figure 2) will be transported alongside the sample. This enables the measurement of acceleration along three spatial axes with a sampling rate of 1 kHz, storing the highest recorded value at 50 Hz. Owing to its lightweight and miniaturized design, the sensor can be easily transported within the PTS. The digital analysis of recorded data allows visualization of acceleration throughout transport (Figure 3A). The forces acting during transport can be graphically represented as the area under the curve (AUC) value for each transport instance (Figure 3C). Specifically, acceleration during transport is quantified by calculating the AUC, with a modification to the approach of Streichert et al. (28). This involves classifying absolute vector values into bins, similar to a histogram (Figure 3B), as described by Streichert et al. (28). The values exceeding a predefined acceleration threshold are subsequently summed (cutoff in Figure 3C). The threshold acceleration is defined as the typical acceleration occurring during manual transport. In contrast to Streichert et al. (28), the AUC calculation in the VETaPT trial involves dividing the result by the accelerometer’s sampling rate. This adjustment ensures generalizability, as otherwise, the AUC would be dependent on the sampling rate of the accelerometer. The AUC is calculated as follows:


$$\mathrm{AUC}=\frac{1}{f}*\sum_{i_{0}}n_{i}*\frac{\left(a_{u,i}+a_{l,i}\right)}{2}$$

where f represents the accelerometer sampling rate and where i_0_ denotes the index of the first bin exceeding the acceleration threshold. Within the sum operator, for each bin i, the product of the number of counts (n_i_) and its mean acceleration, which is calculated as the mean of the upper (a_u,i_) and lower (a_l,i_) bin edges, are multiplied. The formula provided above yields results equivalent to numerically integrating the pure acceleration curve (Figure 3A) while considering only accelerations exceeding the chosen threshold. This approach enables the correlation of differences in coagulation test results between transport methods with their respective AUC values. Consequently, the assumed relationship between acceleration forces and coagulation test results should become evident across different acceleration levels and PTS compressor settings. This method replaces the commonly used yet imprecise characterization of acting forces on the basis of average transport speed with a more methodologically robust and physically accurate approach. By doing so, the acceleration forces—one of the key parameters influencing coagulation activation—are quantified in a standardized and comparable manner. The measured AUC value thereby serves as a parameter that captures the total magnitude of forces exerted by the PTS.

### 2.7 Diagnostic methods

#### 2.7.1 Point-of-care devices for coagulation diagnosis

A wide range of coagulation tests are available for point-of-care diagnostics, with different manufacturers offering similar test principles, as well as various methods that capture different aspects of coagulation. Among them, viscoelastic testing principles play a prominent role. Essentially, all POC VET devices measure the viscoelastic properties of blood clots formed in whole blood samples. By quantifying the kinetics of clot formation and the strength of the clot, these devices enable the detection and algorithm-driven treatment of coagulation disorders. In the context of the VETaPT trial, various POC hemostasis testing systems are employed. While the viscoelastic testing devices operate on the shared principle of analyzing the clot’s mechanical properties during formation, they differ in technical implementation and the reagents used to initiate coagulation. In contrast, the Multiplate^®^ analyzer is based on a different principle: it measures platelet function by detecting changes in electrical impedance as activated platelets adhere and aggregate on sensor wires. This approach represents a fundamentally different methodology from viscoelastic testing. Owing to the point-of-care nature of all these devices, the results are available at the site of critical care in a timely manner.

#### 2.7.2 ClotPro^®^

The ClotPro^®^ (Haemonetics, United States) uses a cuvette with 340 μL of whole blood, where coagulation is initiated by adding coagulation factors. The cuvette is sealed with a plunger. As the measurement begins, the cuvette starts to rotate. The clot that forms between the cuvette and the plunger slows the rotation, creating a resistance that is graphically displayed and output as the clot strength in millimeters. The addition of different agents into the cuvette with coagulation factors changes the results of the ClotPro^®^ test. Currently, nine different test approaches exist for the ClotPro^®^ system, although not all are part of the VETaPT trial. These nine tests are implemented practically via nine different pipette tips, each containing different medication additives, depending on the test. For example, Fib-Test-specific additives inhibit platelet activity, resulting in a clot composed mostly of fibrin, and this test allows for inferences regarding the fibrinogen content of the patient’s blood. The varying test approaches allow for tailored, factor-specific therapy.

#### 2.7.3 ROTEM^®^ sigma

The ROTEM^®^ sigma (Werfen TEM Innovations, Munich) consists of a cartridge system that contains the cups and pins for different tests, running simultaneously. In this approach, the cups are stationary while the pins are moved. An optical system detects displacement via a change in the reflection of a laser so that, knowing the applied force, the deceleration can be determined. It is then also graphically displayed and output as the clot strength in millimeters. For ROTEM^®^ sigma, two test cartridges are available, each containing four running tests. The “ROTEM^®^ sigma complete+hep” cartridge includes the following tests: FIBTEM, EXTEM, INTEM, and HEPTEM. The latter inhibits any potential heparin effect. The “ROTEM^®^ sigma complete” cartridge includes the tests FIBTEM, EXTEM, INTEM, and APTEM. The latter detects potential hyperfibrinolysis through the tranexamic acid included in the test.

#### 2.7.4 TEG6s^®^ and TEG^®^ PlateletMapping

The TEG6s® system (Haemonetics, United States) is also a cartridge system. The determination of clot thickness is no longer mechanical, as it is based on the delay of a moving component but rather involves the measurement of the resonance frequency of the blood sample, which correlates with the viscoelastic properties of the developing thrombus. A solid clot, for example, has a significantly higher resonance frequency than uncoagulated, liquid whole blood. Since the resonance frequency corresponds to the largest amplitude of mechanical displacement of the clot, the resonance frequency can be determined by its measurement via a diode. The determined frequency is then translated into a VET curve by converting it into a clot thickness, also given in millimeters. TEG® PlateletMapping is a cartridge for the TEG6s® system that includes viscoelastic tests with the platelet activators adenosine diphosphate (ADP) and arachidonic acid (AA). This allows for the calculation of the proportion of drug-inhibited platelets.

#### 2.7.5 Multiplate^®^

Multiplate^®^ (Roche Diagnostics, Basel, Switzerland) is a system for platelet aggregometry that electrically measures platelet activation after the addition of the following initiators: ADP, AA, and thrombin receptor-activating peptide 6 (TRAP). The adhesion of activated platelets to the measuring electrodes leads to a change in impedance, which is measured relative to a reference electrode. The use of multiple electrodes allows for internal quality control within each measurement.

The following laboratory, patient, and participant data will be collected as part of the study:

- *Conventional laboratory parameters for hemolysis detection:*
  Blood count, potassium, lactate dehydrogenase (LDH), aspartate aminotransferase (AST), haptoglobin, hemolysis index
- *Conventional laboratory coagulation parameters:*
  International normalized ratio (INR), activated partial thromboplastin time (aPTT), thrombin time, fibrinogen
- *ClotPro^®^:*

ExTest, InTest, FibTest, tPATest, ECAtest, and RVVtest with the following parameters estimated:

- Clotting Time (CT)
- Clotting Formation Time (CFT)
- Angle *α*
- Clot firmness after 5 min (A5)
- Clot firmness after 10 min (A10)
- Maximum Clot Firmness (MCF)
- Maximum Fibrinolysis (ML)
- Lysis time (LT)
- *ROTEM^®^ sigma:*

EXTEM, INTEM, FIBTEM, APTEM – with the following parameters estimated:

- Clotting Time (CT)
- Clotting Formation Time (CFT)
- Clot firmness after 5 min (A5)
- Clot firmness after 10 min (A10)
- Maximum Clot Firmness (MCF)
- Maximum Fibrinolysis (ML)
- Lysis time (LT)
- *TEG6s^®^*:

CK, CKH, CRT, and CFF with the following parameters estimated:

- Reaction Time (R)
- Clotting speed (K)
- Angle
- Maximum Clot Firmness (MA)
- Clot Stability (Lyse30)
- *TEG^®^ PlateletMapping* with the following parameters estimated:
- Maximum Clot Firmness after Kaolin with Heparinase (HKH-MA)
- Maximum Clot Firmness after Activator F (ActF-MA)
- Maximum Clot Firmness after Activator F with Arachidonic Acid (AA-MA)
- Maximum Clot Firmness after Activator F with ADP (ADP-MA)
- Percentage of ADP-inhibited platelets (ADP % Inhibition/% Aggregation)
- Percentage of AA-inhibited platelets (AA % Inhibition/% Aggregation)
- *Multiplate^®^:*
- ADPtest
- ASPItest
- TRAPtest
- *Demographic and disease-specific data of critically ill septic patients:*

**Table 1:** 

Date of admission	Date, time
Date of blood collection	Date, time
Date of birth	Date
Height (cm)	Number
Weight (kg)	Number
Gender	m/f/d
Main diagnosis	Text
SAPS-II-Score	Number
APACHE-II-Score	Number
SOFA-Score Zahl	Number
Charleston-Comorbidity-Index	Number

**Table 2:** 

Comorbidities	
Arterial hypertension	Y/N
Diabetes	Y/N
Obesity	Y/N
Coronary heart disease	Y/N
Chronic arrhythmia	Y/N
Chronic obstructive lung disease	Y/N
Other lung diseases	Y/N
History of venous thrombosis	Y/N
Nicotine abuse	Y/N
History of organ or bone marrow transplant	Y/N
Chronic kidney disease	Y/N
Chronic dialysis	Y/N
Bleeding disorder on admission	Y/N
Thromboembolic complications on admission	Y/N
History of ischemic stroke	Y/N
History of hemorrhagic stroke	Y/N
Acute COVID-19 infection	Y/N
Date of positive COVID test	Date
History of COVID-19-infection	Y/N
Nosocomial infection	Y/N
COVID-19 test on admission	Y/N

**Table 3:** 

Current medication at time of blood sampling	
ACE inhibitor	Y/N
AT2 receptor blocker	Y/N
Beta blockers	Y/N
Corticoids	Y/N
Immunosuppressive medication	Y/N
Platelet inhibition	Y/N
Dual platelet inhibition	Y/N, name of drug, dose
Oral anticoagulation therapy	Y/N, name of drug, dose
Time of last intake	Date, time
Other anticoagulation drugs	Name of drug
Unfractionated heparin	Dose
Low molecular weight heparin	Dose
Other anticoagulation drug	Dose

**Table 4:** 

Condition at time of blood collection	
Ventilated/tracheotomized	Y/N
Glasgow-Coma-Scale	Number
paO_2_	Number
FiO_2_	Number
Blood pressure systolic	Number
Blood pressure diastolic	Number
Blood pressure mean	Number
Heart rate	Number
Catecholamine	Name of drug
Catecholamine dose	Number
Inotropic	Name of drug
Inotropic	Dose
Bilirubin	Number
Thrombocytes	Number
Creatinine	Number
Body temperature	Number
Breathing rate	Number
pH value	Number
Sodium	Number
Potassium	Number
Bicarbonate	Number
Hemoglobin	Number
Hematocrit	Number
Leukocytes	Number
ECMO therapy	Y/N
Date and time of ECMO initiation	Date, time
D-dimer number	Number
Leukocytes	Number
C-reactive protein	Number
Procalcitonin	Number

**Table 5:** 

Complications during the last 24 h before blood sampling	
Bleeding	Y/N, date
Type of bleeding	Text
Blood loss	ml
Blood products, packed red blood cells	Y/N, number
Blood products, fresh frozen plasma	Y/N, number
Blood products, platelet concentrate	Y/N, number
Blood products, 4-factor concentrate	Y/N, number
Blood products, other	Y/N, name, number

- *Demographic data of healthy participants:*

**Table 6:** 

Date of blood collection	Date, time
Date of birth	Date
Size	Number
Weight	Number
Gender	m/f/d

#### 2.7.6 Timeline

The study is planned to run over a total period of 2 years, with a recruitment phase lasting 9 months.

#### 2.7.7 Sample size and statistical analysis

The primary objective of this study is to assess whether the area under the curve (AUC), as a measure of the forces exerted by the pneumatic tube system (PTS), has a clinically significant effect on coagulation analyses with the VET. The parameter used for sample size calculation is the clotting time (CT), a test result of viscoelastic testing. The primary endpoint for the sample size calculation is the binary outcome of a clinically significant change in CT. Clinical significance is defined on the basis of established treatment algorithms used in routine clinical practice (2). In these treatment algorithms, a CT change of ≥20 s in the ClotPro^®^ ExTest significantly influences therapeutic decisions and is thus considered clinically relevant. To enhance the level of safety, in this study a clinically significant difference was defined as a change in CT of ≥10 s between manual transport and PTS transport. A logistic regression model is employed for the sample size calculation, with the binary outcome of a clinically significant change as the dependent variable. We plan to perform the analysis after the completion of the study via logistic regression as well. The independent variables will be the AUC in both cohort (healthy participants vs. critically ill septic patients). Owing to the lack of prior data for sample size planning via ClotPro^®^, estimates will be derived from published data on ROTEM^®^. Given the similar underlying principles of both devices, these data can reasonably be applied for sample size estimation.

The sample size calculation is based on estimating the probability of the primary endpoint under two conditions (H_0_ and H_1_). Since direct probability estimates for H_0_ and H_1_ are unavailable, literature data for CT are used:

- H₀ (Null Hypothesis): The probability of a clinically relevant CT change when the AUC is at its mean value, i.e., manual transport, where no significant effect is expected. The probability of a clinically relevant CT change due to manual transport is considered very low. On the basis of Martin et al. (10), this probability is estimated at 0.045.
- H₁ (Alternative Hypothesis): The probability of a clinically relevant CT change when the AUC is at its mean plus one standard deviation, i.e., PTS transport, where an effect is expected. The probability of a clinically relevant CT change due to increased AUC in PTS transport can be inferred from the method established by Streichert et al. (28). On the basis of the mean difference and standard deviation of CT changes between transport methods in Martin et al. (10), this probability is estimated at 0.21.

Sample size calculation was performed via G-Power 3.1 (29) with the following parameters: significance level (*α*) = 0.05; power (1-*β*) = 0.8; two-sided test; R^2^ other X = 0.36; Wald’s Z test. The total required sample size was determined to be N = 83 paired comparisons (manual vs. PTS transport). Considering potential dropouts due to sampling errors, withdrawal of consent, or failures in point-of-care testing, a dropout rate of 10% is assumed, increasing the total required sample size to 92. The participants will be equally distributed between the two study groups, resulting in 46 cases per group (e.g., healthy volunteers and critically ill septic patients).

Baseline comparisons between the two cohorts will be conducted via t tests for normally distributed data and the Wilcoxon test when normality is violated. Normality will be assessed via the Shapiro–Wilk test. A *p* value ≤ 0.05 was considered statistically significant. All the statistical analyses will be performed via MATLAB software (The MathWorks Inc., 2024).

## 3 Discussion

Previous findings on the impact of PTS commonly used in hospitals on VET results are inconsistent (12, 13) and may not be applicable to next generation VET devices (2, 14, 16). This study aims not only to determine whether PTS exerts a clinically significant influence on VET results but also to identify factors contributing to potential alterations. The following aspects are considered:

- *VET-related factors*: a comparison of next generation VET devices;
- *PTS-related factors*: assessed by continuously quantifying acceleration forces;
- *Patient-related factors*: analyzed by comparing healthy volunteers with sepsis patients;

This study represents the first to allow such a differentiated approach within a single study design. By utilizing this design, future research can facilitate the transferability of findings to other PTS systems by measuring the acceleration forces acting on transported samples, independent of manufacturer-reported transport speeds. Furthermore, the comparison of healthy volunteers with sepsis patients in this study aims to provide insights into whether pathological coagulation activation in sepsis patients alters susceptibility to acceleration forces. These findings may help explain the discrepancies reported in previous studies, which focused on either healthy individuals or patient cohorts (9, 10, 22, 23). The sub-experiment in this study employing a vibration exciter enables differentiation between the effects of absolute acceleration magnitudes and continuous exposure to acceleration forces on the VET results. These insights could form the basis for further investigations into coagulation activation in extracorporeal circulation settings, where both shear stress and flow acceleration occur.

A study-related limitation is the lack of blinding of investigators during the initiation of VET measurements. However, as VET measurements follow a standardized protocol, the impact of this limitation is considered minimal. Another limitation is the inability to assess intra-device measurement variability, i.e., the precision of the VET devices. Due to the necessity in this study of multiple measurements under both transport conditions and the maximum sample capacity of the devices, additional measurements to evaluate device precision could not be performed within the predefined time frame after blood collection.

This study is the first to simultaneously compare multiple next generation VET devices while distinguishing between healthy volunteers and sepsis patients and to conduct precise quantification of acceleration forces during PTS transport.

The findings of this study can contribute to the safe application of PTS for the transport of blood samples for VET analyses. This allows for faster transport with reduced personnel requirements, leading to quicker results and earlier initiation of therapies. Moreover, these findings may serve as a foundation for further studies on coagulation activation in extracorporeal circulation settings.

## 4 Data management

### 4.1 Documentation

All data collected during the study, including medical history, comorbidities, test results, and adverse events, will be recorded by authorized personnel. A special identification list will be maintained to ensure the identification of patients and participants after the study has been completed. If a participant withdraws consent, they will be removed from the identification list to ensure data privacy and confidentiality.

### 4.2 Data collection

Patient data will be collected via the patient data management system of the intensive care unit of the study center. Data collection from healthy volunteers will be performed both by using the patient data management system of the hospital of the study center for collecting laboratory and point-of-care data and by filling a data template for biometric data. Data collection will be carried out in accordance with the established study methods, and all collected data will be treated in compliance with data protection regulations.

### 4.3 Withdrawal of individual participants (dropout)

If the exclusion criteria are unknowingly met at the time of study enrollment, later exclusion may occur. Withdrawal of consent will result in the exclusion of the collected data. Published data are untraceable once published.

### 4.4 Data privacy and confidentiality

Data are collected pseudonymously, meaning that identification can only occur via the identification list. Access to this list will be provided only to those involved in this clinical trial. Participants can withdraw their consent at any time. The raw data will be stored for at least 10 years in accordance with the “Statute for Ensuring Good Scientific Practice” of the TUD Dresden, Dresden, Germany.

### 4.5 Archiving of original data

The collected data are stored in an electronic database within the network of the University Hospital Dresden. The data were deleted no earlier than 10 years after the study’s conclusion.

### 4.6 Access to data

The data collected as part of this study will be made available in pseudonymized form in the Open Access Repository and Archive for Research Data of Saxon Universities OPARA (30). The results of the study are expected to be published.
